# Systemic immune and coagulation amplifiers resolve diagnostic ambiguity in endometriosis-associated ovarian cancer

**DOI:** 10.1186/s13048-026-02205-8

**Published:** 2026-07-23

**Authors:** Li Luo, Yanwei Tu, Zirui Zhu, Mingzhu Ye

**Affiliations:** 1 Department of Obstetrics and Gynecology, Zhongshan Hospital of Xiamen University, School of Medicine, Xiamen University, Xiamen, China; 2 School of Medicine, Xiamen University, Xiamen, China

**Keywords:** Endometriosis-associated ovarian cancer (EAOC), Fibrinogen-to-albumin ratio (FAR), Absolute monocyte count (AMC), Predictive model, Nomogram, Differential diagnosis, Systemic inflammation, Coagulation

## Abstract

**Background:**

Preoperative discrimination between endometriosis-associated ovarian cancer (EAOC) and benign ovarian endometrioma (OE) remains challenging because imaging findings overlap and CA125 is frequently elevated by benign pelvic inflammation. We hypothesized that EAOC is associated with a systemic immune-coagulation signature that could improve risk stratification beyond tumor markers alone.

**Methods:**

We retrospectively analyzed treatment-naive patients who underwent primary surgery for ovarian masses between January 2015 and February 2025. EAOC (*n* = 49) and OE (*n* = 146) were confirmed histopathologically according to the Sampson–Scott criteria. Preoperative laboratory indices obtained within 2 weeks before surgery included fibrinogen, albumin, absolute monocyte count (AMC), CA125, and HE4; the fibrinogen-to-albumin ratio (FAR) was calculated. A multivariable logistic regression model integrating age, menopausal status, AMC, FAR, CA125, and HE4 was developed as the CO-MHF nomogram. Supplementary data-driven analyses included least absolute shrinkage and selection operator (LASSO) logistic regression, Akaike information criterion (AIC)-based stepwise selection, and add-one/drop-one marginal contribution analysis. Discrimination, calibration, and clinical utility were assessed by area under the receiver operating characteristic curve (AUC), bootstrap-derived confidence interval (CI), the Hosmer-Lemeshow test, and decision curve analysis (DCA).

**Results:**

FAR and AMC were significantly higher in EAOC than in OE (median FAR 0.10 versus 0.07; median AMC 0.39 versus 0.31 × 10^9^/L). The CO-MHF model showed favorable internal discrimination (AUC 0.932, 95% CI 0.887–0.968), outperforming HE4 (0.835), FAR (0.786), CA125 (0.769), and AMC (0.657). At the Youden-derived threshold (0.468), sensitivity was 75.5% and specificity 95.9%, with a positive predictive value (PPV) of 86.0% and a negative predictive value (NPV) of 92.1%. Lower thresholds increased sensitivity to 85.7%–93.9% at the cost of reduced specificity. Supplementary data-driven analyses supported the predictor set: AIC-based stepwise selection identified the same variables, and LASSO retained all CO-MHF predictors. Calibration was acceptable (Hosmer–Lemeshow *P* = 0.225), and DCA showed greater net benefit across a range of threshold probabilities.

**Conclusions:**

A mechanism-informed nomogram combining FAR and AMC with established tumor markers may improve preoperative risk stratification for EAOC. This host-response framework merits further evaluation in other malignancy-versus-inflammation diagnostic settings, although prospective multicenter validation and clinical threshold optimization remain necessary.

**Supplementary Information:**

The online version contains supplementary material available at https://doi.org/10.1186/s13048-026-02205-8.

## Introduction

Ovarian cancer is a highly lethal gynecologic malignancy characterized by late clinical presentation and a lack of effective early screening strategies [1]. Substantial histological evidence suggests that endometriosis-associated ovarian cancer (EAOC) is a distinct entity, arising from the malignant transformation of benign endometriotic lesions driven by chronic inflammation [2]. Moreover, preoperative differentiation between EAOC and benign ovarian endometrioma (OE) remains a substantial challenge, as current diagnostic modalities, including pelvic imaging and serum tumor markers, often fail to provide definitive risk stratification [3]. While ultrasonography and magnetic resonance imaging are standard diagnostic tools, morphological overlap often leads to inconclusive findings [4]. Furthermore, this diagnostic ambiguity is compounded by the limitations of conventional serological biomarkers [5].

Carbohydrate antigen 125 (CA125) demonstrates compromised diagnostic utility in endometriosis. Peritoneal irritation often triggers false positive elevations mimicking malignancy, leaving significant diagnostic gaps [6]. Furthermore, while human epididymis protein 4 (HE4) offers improved specificity, relying on single or dual biomarkers leaves a residual diagnostic gap. This gap is further complicated by the emerging recognition that EAOC comprises heterogeneous clinicopathological entities. Recent studies have distinguished endometriosis-correlated ovarian carcinoma, defined by histopathological transition from endometriosis to carcinoma, from endometriosis-incidental ovarian carcinoma, in which endometriosis coexists without identifiable transitional lesions [7, 8]. These subtypes differ in disease stage, histological distribution, molecular-pathological features, and clinical behavior, indicating that single tumor-antigen markers may be insufficient to capture the full biological spectrum of EAOC. This supports the need for a composite preoperative model integrating conventional tumor markers with systemic host-response indicators [9, 10]. Beyond conventional diagnostic approaches, the transition from benign endometriosis to malignancy is associated with disruption of host immune and coagulation homeostasis [11]. Specifically, hypercoagulability involves upregulated fibrinogen. This protein acts as both a clotting factor and a molecular scaffold that facilitates tumor cell adhesion and shields circulating cancer cells from immune surveillance [12].

Empirical findings demonstrate that declining serum albumin reflects compromised host nutritional status and an active systemic inflammatory response during carcinogenesis [13]. This malignant transformation induces a systemic immune and coagulation amplification loop effectively captured by the fibrinogen to albumin ratio (FAR) [14]. Moreover, evidence indicates that cytokines derived from the tumor reprogram hepatic protein synthesis to prioritize fibrinogen production while suppressing albumin [15]. Consequently, the expansion of the absolute monocyte count (AMC) reflects the recruitment of macrophages associated with the tumor [16]. Therefore, FAR and AMC serve as accessible serological proxies for this tumorigenic process. These markers may therefore complement conventional tumor markers and improve diagnostic precision in inflammation-confounded diagnostic settings [17].

Therefore, guided by the principles of clinically interpretable prediction modeling [18, 19] and the need to capture ovarian cancer heterogeneity beyond single biomarkers [20], this study aimed to develop and internally evaluate a preoperative nomogram for distinguishing EAOC from benign OE. The CO-MHF model integrated systemic immune-coagulation indicators, including FAR and AMC, with established tumor markers and clinical variables. We further assessed its discrimination, calibration, clinical net benefit, robustness to class imbalance, threshold-dependent diagnostic trade-offs, and empirical support for the final predictor set.

## Methods

### Study design and participants

This retrospective cohort study was conducted at the Department of Gynecology at Zhongshan Hospital of Xiamen University. We reviewed clinical and laboratory data for patients undergoing surgical treatment for ovarian masses between January 2015 and February 2025. The study protocol was approved by the institutional review board and the requirement for informed consent was waived due to the retrospective nature of the analysis.

During the study period, 546 patients underwent surgery for primary ovarian malignancies at our institution, irrespective of histological subgroup, including 482 patients with epithelial ovarian cancer. Among these, 76 patients were histopathologically diagnosed as EAOC according to the Sampson–Scott criteria. These institutional ovarian cancer denominators were reviewed to clarify the source population and to avoid interpreting the final analytical cohort as an estimate of EAOC prevalence among all ovarian cancers.

**Fig. 1:**
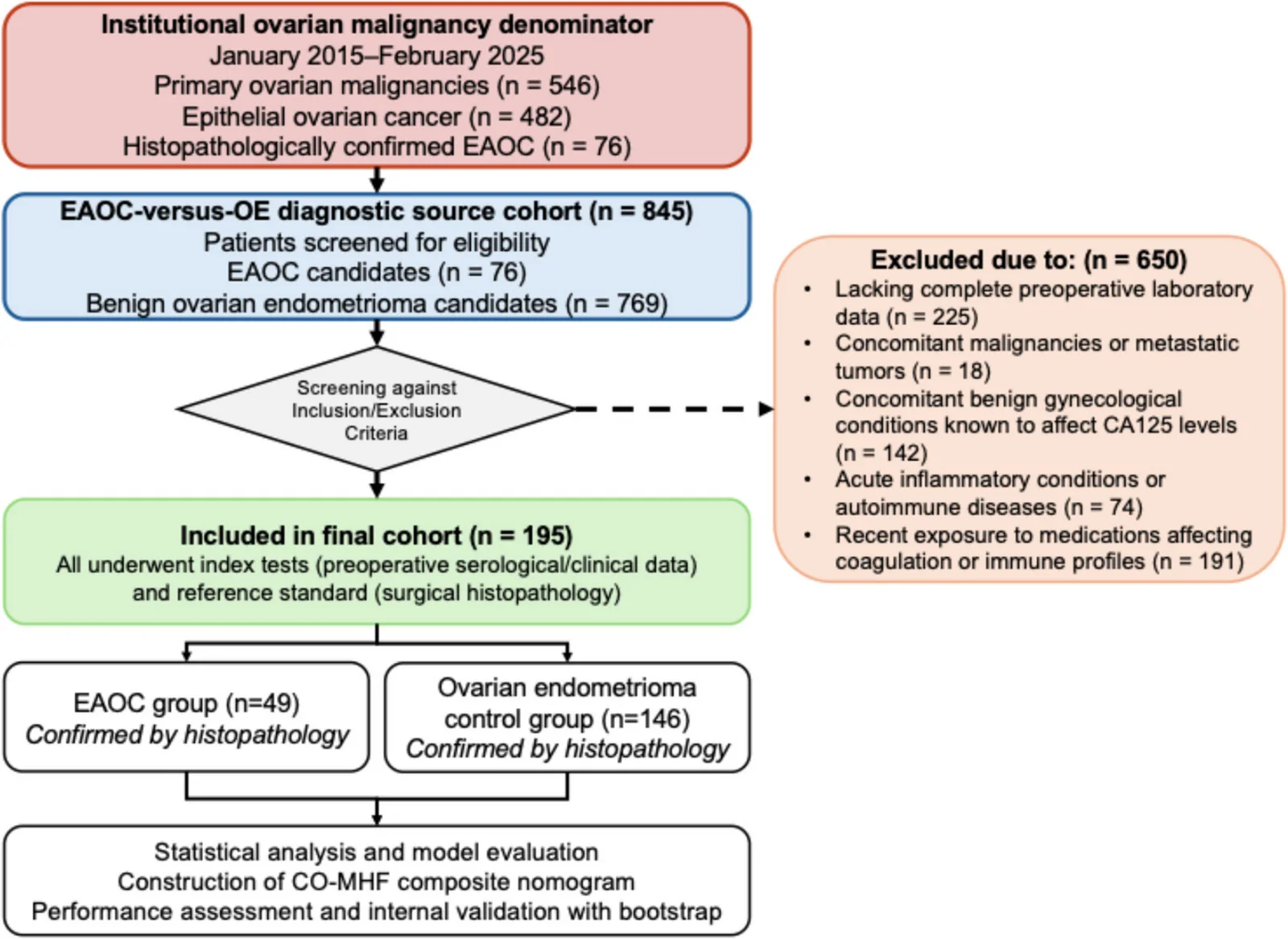
Source population, patient screening, exclusion, and final analytical cohort construction. Institutional ovarian cancer denominators are shown to clarify the background source population. The EAOC-versus-OE diagnostic cohort was constructed for preoperative model development and should not be interpreted as the proportional distribution of ovarian cancer histological subgroups. Exclusion categories included incomplete preoperative laboratory data, concomitant malignancies or metastatic tumors, benign gynecological conditions affecting CA125, acute inflammatory or autoimmune conditions, and recent medication exposure affecting coagulation or immune profiles

For construction of the EAOC-versus-OE diagnostic model, 845 patients with histopathologically confirmed EAOC or benign OE were screened for eligibility, including 76 patients with EAOC and 769 patients with benign OE. A total of 650 patients were excluded according to predefined clinical and laboratory criteria (Fig. 1). These included 225 patients lacking complete preoperative laboratory data, 18 patients with concomitant malignancies or metastatic tumors, 142 patients with concomitant benign gynecological conditions known to affect CA125 levels, 74 patients with acute inflammatory conditions or autoimmune diseases, and 191 patients with recent exposure to medications affecting coagulation or immune profiles. The final analytical cohort included 195 patients, comprising 49 patients with EAOC and 146 patients with benign OE.

Complete preoperative laboratory profiles were required because the CO-MHF model was constructed using simultaneously available coagulation, inflammatory, nutritional, and tumor-marker variables, including fibrinogen, albumin, AMC, CA125, and HE4. Therefore, patients with incomplete profiles for these core predictors were not included in model development. All included patients received their initial surgical intervention at our institution and were treatment-naive before surgery.

No strict individual age matching was performed. The final benign OE control group represented a clinically age-overlapping diagnostic control group for the specific task of distinguishing EAOC from benign OE under complete preoperative laboratory availability, rather than a cohort intended to represent the full epidemiological age distribution of benign OE. Therefore, age was retained as a prespecified clinical covariate in the CO-MHF model.

### Diagnostic standards and selection rigor

Histopathological diagnosis of EAOC followed the classic criteria established by Sampson and Scott [21]. Inclusion required at least one of three established conditions. These included microscopic evidence of a direct transition from benign endometriosis to carcinoma or the coexistence of both within the ipsilateral ovary. Alternatively, the presence of definitive endometriotic lesions in other pelvic sites with histological similarity to the ovarian carcinoma was sufficient. Secondary ovarian carcinomas of other histological origins were excluded. For the control group, histopathology confirmed the diagnosis of benign OE and excluded malignant or borderline components.

All patients had complete preoperative laboratory data obtained within two weeks before surgery. Patients with missing values in any core predictor were excluded rather than imputed. This complete-case strategy was chosen because the missing laboratory variables represented essential components of the CO-MHF model rather than auxiliary covariates. In particular, HE4 availability, and therefore availability of the complete predictor set, was likely influenced by historical testing patterns and clinical diagnostic pathways during the 10-year study period, making the assumption of completely random missingness unlikely. Multiple imputation in this setting could therefore introduce additional uncertainty and potentially unstable estimates in a relatively small retrospective cohort. Metrics included a complete blood count, a coagulation profile for fibrinogen, liver and kidney function tests for albumin, and serum tumor markers CA125 and HE4. The FAR was calculated as the serum fibrinogen level divided by the serum albumin level [14]. The neutrophil-to-lymphocyte ratio (NLR) was calculated as the absolute neutrophil count divided by the absolute lymphocyte count and was included only in the supplementary data-driven candidate predictor assessment. Furthermore, a multidimensional exclusion strategy eliminated potential confounders of the systemic immune and coagulation profiles. We excluded patients with previous malignancies, metastatic ovarian tumors, or benign conditions such as adenomyosis and uterine fibroids that affect CA125 levels. Patients with acute systemic or genitourinary infections within two weeks before surgery, chronic inflammation, or autoimmune diseases were excluded to avoid monocyte count alterations. Individuals with primary hematological disorders or severe organ insufficiency affecting the synthesis and excretion of albumin and tumor markers were also excluded. Finally, we excluded pregnant or lactating women and patients with prior exposure to radiotherapy, chemotherapy, or immunotherapy. We also excluded patients receiving hormonal therapies, immunosuppressants, antibiotics, or medications affecting coagulation within three months before blood collection.

### Statistical analysis

Statistical analyses were performed to evaluate the diagnostic framework and construct the predictive scoring system. Continuous variables with non-normal distributions were expressed as medians with interquartile range (IQR) and compared using the Mann–Whitney U test. We developed the primary CO-MHF nomogram using multivariable logistic regression incorporating age, menopausal status, AMC, HE4, CA125, and FAR. These predictors were prespecified on the basis of clinical relevance and biological plausibility to preserve interpretability and consistency with the proposed immune–coagulation host-response framework.

To examine whether the prespecified predictors were also supported empirically by the available complete-case data, we performed supplementary data-driven candidate predictor assessments. Because the final model was designed as a calibrated and clinically interpretable logistic-regression-based nomogram, random forest-based feature importance was not used as a formal variable-selection procedure. Instead, we used least absolute shrinkage and selection operator (LASSO) logistic regression, Akaike information criterion (AIC)-based stepwise logistic regression, and add-one/drop-one marginal contribution analysis. These approaches were chosen because they are directly aligned with the logistic-regression framework of the final nomogram and allow empirical assessment of predictor relevance while preserving clinical interpretability.

A non-redundant candidate predictor set was constructed to avoid mathematical redundancy and multicollinearity. Candidate variables included age, pregnancy history, menopausal status, AMC, CA125, HE4, FAR, and NLR. Because FAR is derived from fibrinogen and albumin, fibrinogen and albumin were not entered simultaneously with FAR in the primary data-driven candidate set. Continuous variables were standardized before LASSO regression. The optimal penalty parameter was selected using stratified five-fold cross-validation, and the one-standard-error rule was also examined to assess model parsimony. AIC-based stepwise logistic regression was used to identify the candidate predictor subset that balanced model fit and complexity. Add-one and drop-one analyses were then performed to quantify the marginal contribution of retained and excluded candidate variables by examining changes in area under the receiver operating characteristic curve (AUC) and AIC.

Given the moderate imbalance between EAOC and OE cases, artificial over-sampling or under-sampling was not applied in the primary model because the objective was to develop a clinically interpretable diagnostic risk model with preserved probability calibration rather than a purely classification-oriented algorithm. Artificial resampling may alter the intercept and distort absolute risk estimation, which is particularly relevant for a nomogram intended for individualized preoperative risk assessment. To evaluate whether the observed class distribution materially influenced model performance, we additionally performed a sensitivity analysis using class-weighted logistic regression with the same predictors.

Bootstrap resampling with 1000 iterations was used to estimate the uncertainty of model performance metrics. To ensure mathematical transparency and facilitate independent external validation, the probability of malignancy * P* was computed utilizing the standard logistic regression formula $$\:P=1/\left(1+{e}^{-Z}\right)$$, where * Z* represents the linear predictor. The generalized multivariable risk algorithm was structured as $$\:Z={\beta\:}_{0}+{\beta\:}_{1}\times\:\mathrm{Age}$$$$+{\beta\:}_{2}\times\:\mathrm{Menopause}+{\beta\:}_{3}$$ $$\times\:\mathrm{AMC}+{\beta\:}_{4}\times\:\mathrm{HE4}+{\beta\:}_{5}$$$$\times\:\mathrm{FAR}+{\beta\:}_{6}\times\:\mathrm{CA125}$$ , with $$\:{\beta\:}_{0}$$ functioning as the baseline intercept and the subsequent coefficients representing the independent mathematical weighting of each specific variable. The finalized specific equation alongside the derived intercept and constant values is detailed in the results section. The diagnostic performance of the composite model and individual biomarkers was assessed by calculating the AUC. The primary accuracy-optimized cut-off value was determined using the Youden index to calculate sensitivity, specificity, accuracy, positive predictive value (PPV), and negative predictive value (NPV). In addition, sensitivity-prioritized thresholds were explored to characterize the clinical trade-off between false-negative reduction and specificity loss. These additional thresholds were used for interpretative purposes and did not replace the primary Youden-derived threshold. The 95% confidence interval (CI) for evaluated metrics was generated through 1000 bootstrap resampling iterations. The DeLong test was applied to evaluate differences between correlated receiver operating characteristic (ROC) curves. The clinical reliability of the nomogram was evaluated utilizing a calibration curve alongside the Hosmer-Lemeshow goodness of fit test and the mean absolute error. Decision curve analysis (DCA) was conducted to quantify the clinical net benefit of the composite model across a range of threshold probabilities. All statistical analyses were performed using Python 3.9.18 with pandas 2.2.2, NumPy 1.26.4, SciPy 1.13.1, statsmodels 0.14.2, scikit-learn 1.5.1, matplotlib 3.8.4, and seaborn 0.13.2. A two-sided P value of less than 0.05 was considered statistically significant.

## Results

### Baseline characteristics of the study population

During the study period, 546 patients underwent surgery for primary ovarian malignancies at our institution, including 482 patients with epithelial ovarian cancer. Among them, 76 patients were histopathologically diagnosed as EAOC according to the Sampson–Scott criteria. For the EAOC-versus-OE diagnostic cohort, 845 patients were screened, including 76 patients with EAOC and 769 patients with benign OE. After applying the predefined eligibility criteria and complete preoperative laboratory-profile requirement, 195 patients were included in the final analytical cohort, corresponding to an inclusion rate of 23.1%. A total of 650 patients were excluded before final cohort construction, with incomplete preoperative laboratory data representing the most frequent exclusion category (*n* = 225, 26.6% of the screened cohort). The source population, diagnostic cohort construction, and proportional distribution of exclusion categories are summarized in Supplementary Table S1.

This retrospective cohort study enrolled 195 eligible patients, comprising 49 patients diagnosed with EAOC in the malignant group and 146 patients diagnosed with benign OE in the control group. The median age of patients in the malignant cohort was 48.0 years with an IQR of 43.0–53.0 years, which was comparable to the median age of 46.5 years with an IQR of 44.0–49.0 years observed in the benign cohort. The Mann-Whitney U test revealed no significant difference in age distributions between the two groups.

### Validation of the systemic amplification loop

**Fig. 2:**
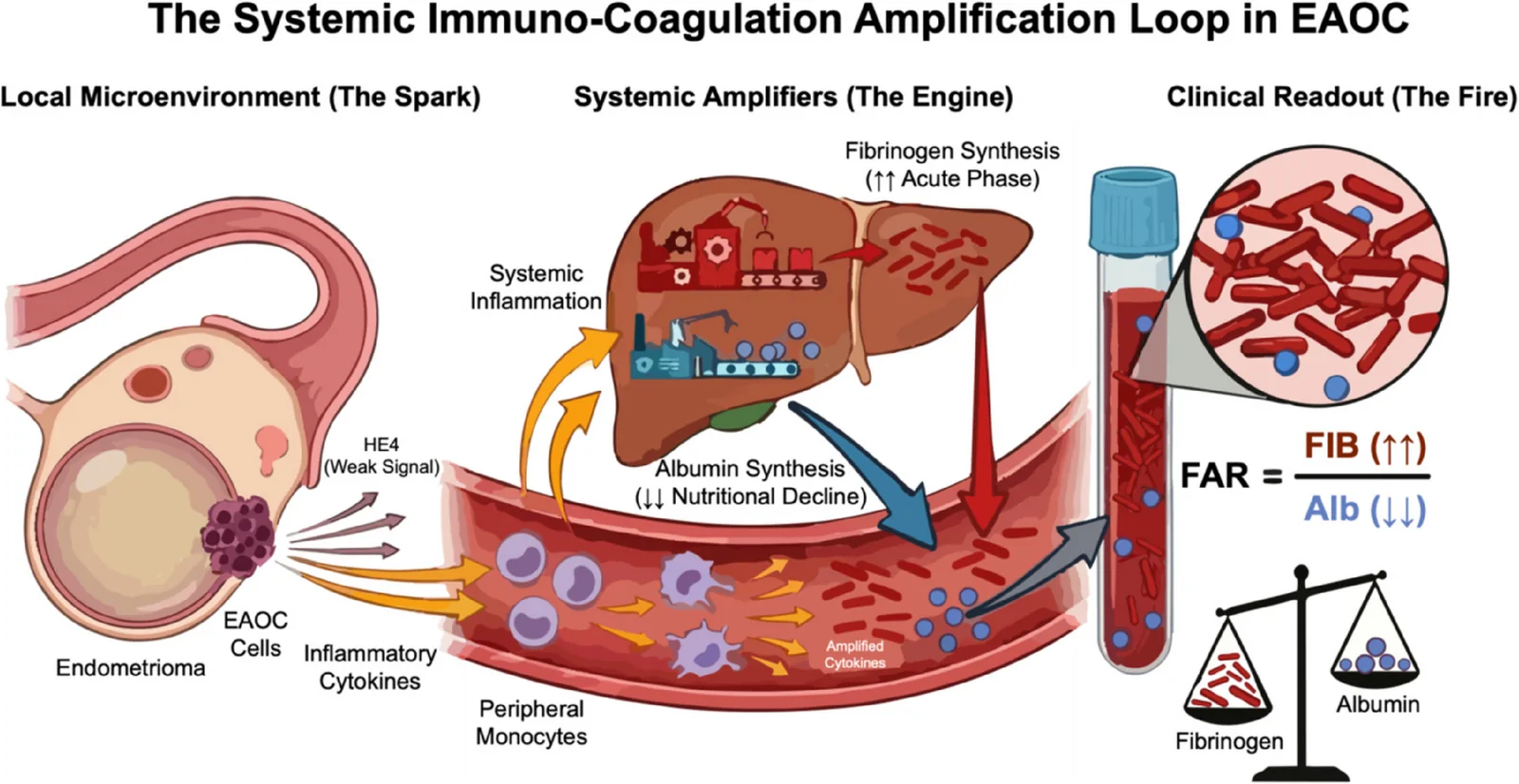
Schematic representation of the systemic immune and coagulation amplification loop. Malignant transformation drives hepatic reprogramming to prioritize fibrinogen synthesis and accelerate albumin consumption while simultaneously recruiting tumor-associated macrophages

**Fig. 3:**
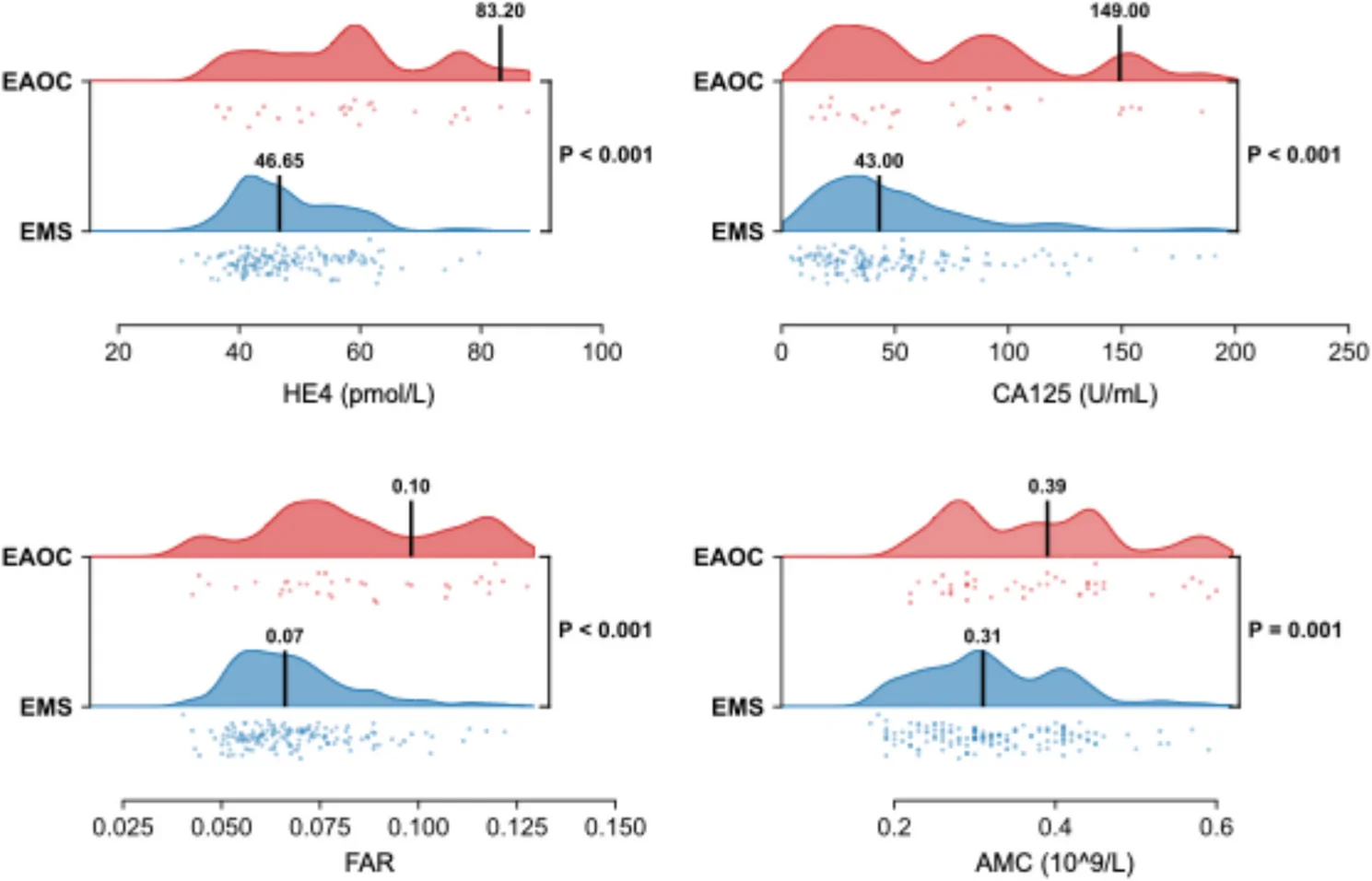
Comparative analysis of preoperative serological indices between the malignant and benign cohorts. The systemic divergence highlights a hypercoagulable and catabolic phenotype alongside expanded absolute monocyte counts in patients with cancer

The biological framework of the systemic immune and coagulation amplification loop is illustrated as a mechanistic model (Fig. 2). Preoperative serological profiles, including the expression levels of evaluated markers, were compared between the EAOC and OE cohorts (Fig. 3). The median FAR was 0.10 in the cancer group compared with 0.07 in the OE group. Concurrently, the AMC was higher in patients with malignancy, presenting a median of 0.39 billion per liter versus 0.31 billion per liter in those with benign OE. The median HE4 level in the cancer group was 83.20 picomoles per liter, exceeding that in the benign group at 46.65 picomoles per liter. Similarly, CA125 levels were 149.00 units per milliliter in the malignant cohort versus 43.00 units per milliliter in the control cohort.

### Construction and performance of the CO-MHF model

A multivariable logistic regression analysis incorporated the immune and coagulation indices with conventional tumor burden markers to construct the CO-MHF composite model. Standardized coefficients were extracted to determine the relative statistical contribution of each predictor, including menopausal status, age, CA125, HE4, FAR, and AMC (Supplementary Fig. 1). Menopausal status and CA125 yielded standardized coefficients of 1.52 and 1.50, respectively. HE4 and FAR provided coefficients of 1.18 and 1.03, respectively. AMC yielded a coefficient of 0.47, while age exhibited a coefficient of -0.94.

Following statistical optimization on the primary analytical cohort, the final linear predictor was: $$\:\mathrm{Z}=4.5547-0.2389\times\:\mathrm{Age}$$$$+4.6324\times\:\mathrm{Menopause}+3.7637$$$$\times\:\mathrm{AMC}+0.0100\times\:\mathrm{HE4}+20.7581$$$$\times\:\mathrm{FAR}+0.0063\times\:\mathrm{CA125}$$ .

To further assess empirical support for the prespecified CO-MHF predictor set, we performed supplementary data-driven candidate predictor assessment using the complete analytical cohort. In the non-redundant candidate predictor set, AIC-based stepwise logistic regression selected age, menopausal status, AMC, CA125, HE4, and FAR, which was identical to the CO-MHF predictor set. LASSO logistic regression with cross-validation also retained all CO-MHF predictors under both the minimum-error model and the one-standard-error rule. NLR was additionally retained by LASSO, but it was not selected by AIC-based stepwise regression. When NLR was added to the CO-MHF model, the AUC increased only slightly from 0.932 to 0.936 and model parsimony did not improve. In drop-one analysis, removing age reduced the AUC from 0.932 to 0.918 and worsened the AIC from 118.44 to 130.07. Removing FAR, HE4, and AMC reduced the AUC to 0.916, 0.923, and 0.927, respectively. Together, these findings supported retention of the final CO-MHF predictor set and suggested that the excluded candidate variables contributed limited additional diagnostic information. The full data-driven predictor-selection and marginal contribution results are summarized in Supplementary Table S2.

**Fig. 4:**
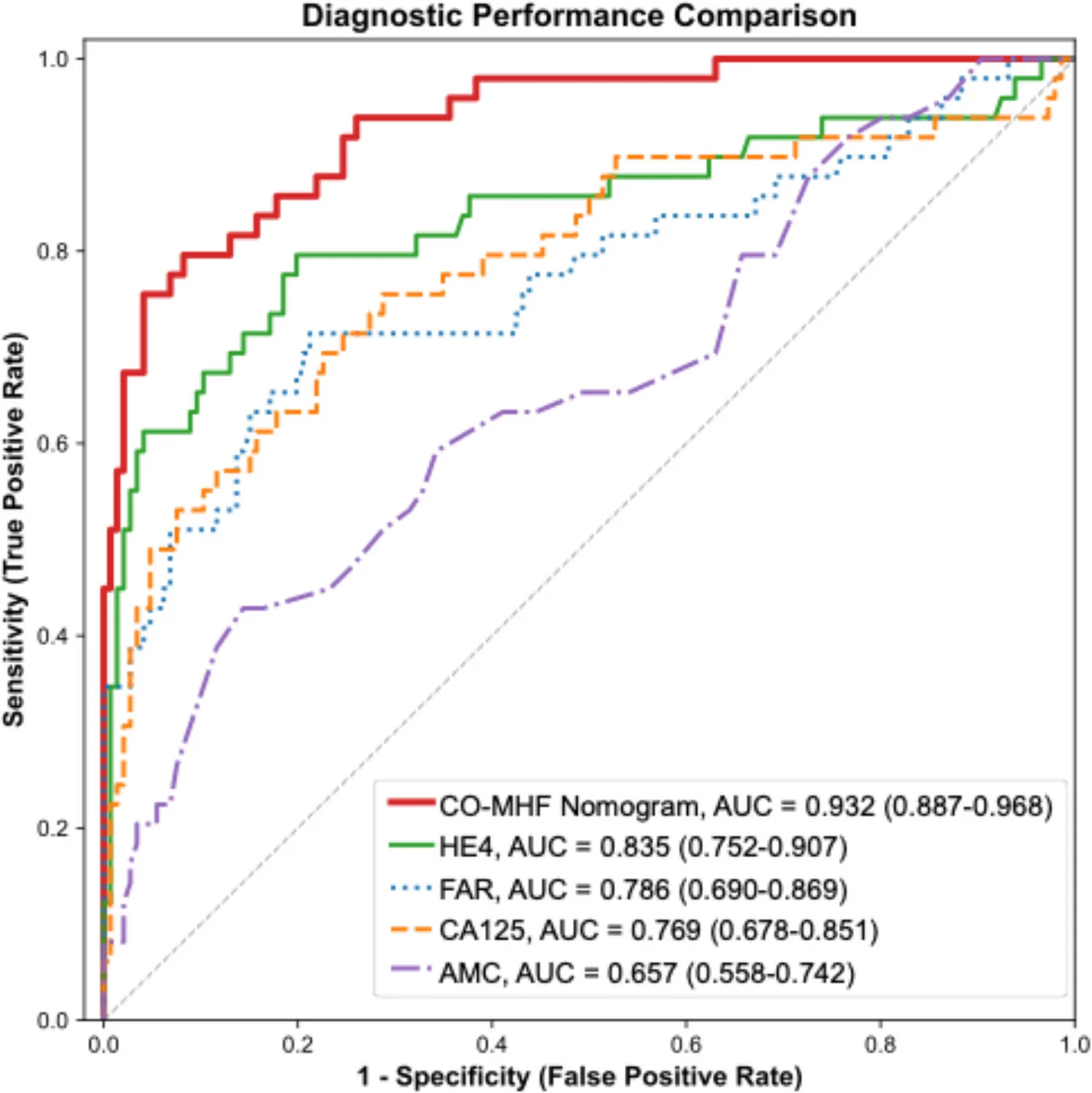
ROC curves comparing CO-MHF with HE4, CA125, FAR, and AMC

The composite model achieved an AUC of 0.932 (95% CI 0.887–0.968) (Fig. 4). In comparison, individual parameters yielded lower AUC values, with HE4 achieving 0.835, FAR achieving 0.786, CA125 achieving 0.769, and AMC achieving 0.657. The primary accuracy-optimized threshold determined using the Youden index was 0.468. At this threshold, the composite model yielded a sensitivity of 75.5% (95% CI 71.9–95.8), a specificity of 95.9% (95% CI 77.9–99.3), and an overall accuracy of 90.8% (95% CI 81.5–96.4). The model achieved PPV and NPV of 86.0% and 92.1%, respectively. A comprehensive summary of these diagnostic metrics across all evaluated markers alongside their 95% CIs derived from 1000 bootstrap iterations is detailed in Table 1.

**Table 1:** Optimal cutoffs and diagnostic performance of CO-MHF and individual biomarkers. The table presents point estimates evaluated at the optimal cut-off point alongside 95% CIs generated through 1000 bootstrap iterations to estimate statistical uncertainty

Biomarker	Cut-off	AUC (95% CI)	Sen% (95% CI)	Spe% (95% CI)	Acc% (95% CI)	PPV%	NPV%
CO-MHF	0.468	0.932 (0.887–0.968)	75.5 (71.9–95.8)	95.9 (77.9–99.3)	90.8 (81.5–96.4)	86.0	92.1
HE4	56.80	0.835 (0.752–0.907)	79.6 (56.6–89.7)	80.1 (76.5–98.1)	80.0 (76.4–90.8)	57.4	92.1
FAR	0.07	0.786 (0.690–0.869)	69.4 (46.2–89.2)	77.4 (57.8–97.2)	75.4 (66.7–88.2)	50.7	88.3
CA125	75.40	0.769 (0.678–0.851)	71.4 (50.0–83.3)	78.8 (73.4–95.7)	76.9 (72.3–86.7)	53.0	89.1
AMC	0.43	0.657 (0.558–0.742)	42.9 (31.1–89.4)	85.6 (32.6–93.3)	74.9 (47.2–81.6)	50.0	81.7

Threshold-dependent analysis showed that lowering the CO-MHF cut-off increased sensitivity at the expense of specificity. At a cut-off of 0.167, the model yielded a sensitivity of 85.7%, specificity of 82.2%, accuracy of 83.1%, PPV of 61.8%, and NPV of 94.5%. At a lower cut-off of 0.099, the sensitivity increased to 93.9%, with a specificity of 74.7%, accuracy of 79.5%, PPV of 55.4%, and NPV of 97.3%. The threshold-dependent diagnostic trade-offs are summarized in Supplementary Table S3.

In the class-weighted logistic regression sensitivity analysis, the model achieved an AUC of 0.936, which was comparable to that of the primary unweighted CO-MHF model (AUC 0.932). At the Youden-derived threshold of the class-weighted model, the sensitivity, specificity, accuracy, PPV, and NPV were 77.6%, 95.9%, 91.3%, 86.4%, and 92.7%, respectively. The detailed comparison between the primary model and the class-weighted sensitivity model is presented in Supplementary Table S4.

### Nomogram construction and clinical calibration

**Fig. 5:**
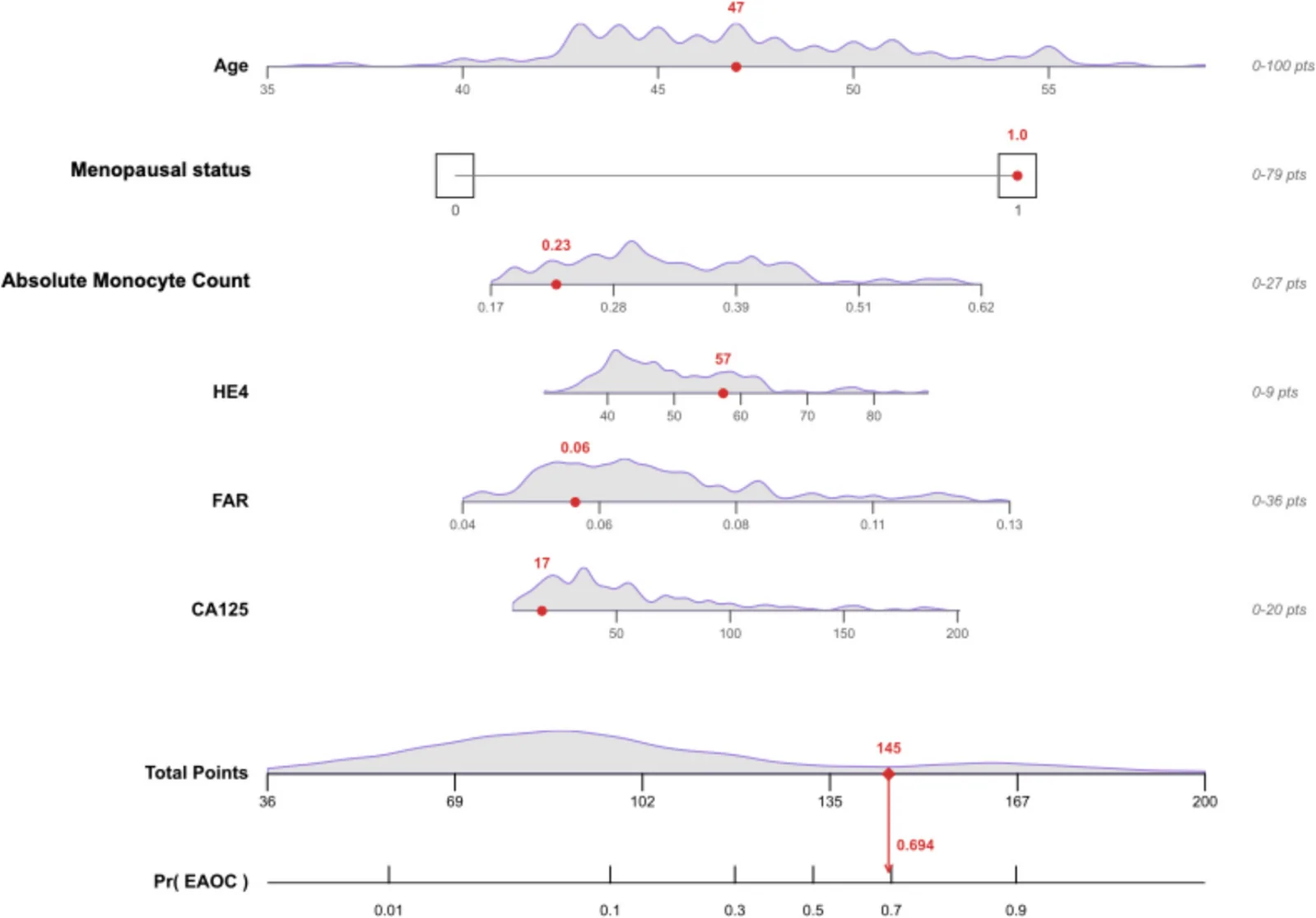
The quantitative nomogram for the individualized preoperative prediction of malignancy probability. Specific point values are assigned to age and menopausal status and the ratio of fibrinogen to albumin alongside absolute monocyte count and conventional tumor markers to calculate a cumulative clinical risk score

A quantitative nomogram (Fig. 5) was constructed to assign point values to each independent predictor based on its regression coefficient. Age was assigned a scale from 0 to 100 points, while menopausal status and FAR contributed maximum scores of 79 points and 36 points, respectively. AMC, CA125, and HE4 provided additional diagnostic weighting with maximum scores of 27 points, 20 points, and 9 points, respectively. Calibration of the scoring system yielded a mean absolute error of 0.198 and a Hosmer-Lemeshow test P value of 0.225.

### Clinical utility and net benefit analysis

**Fig. 6:**
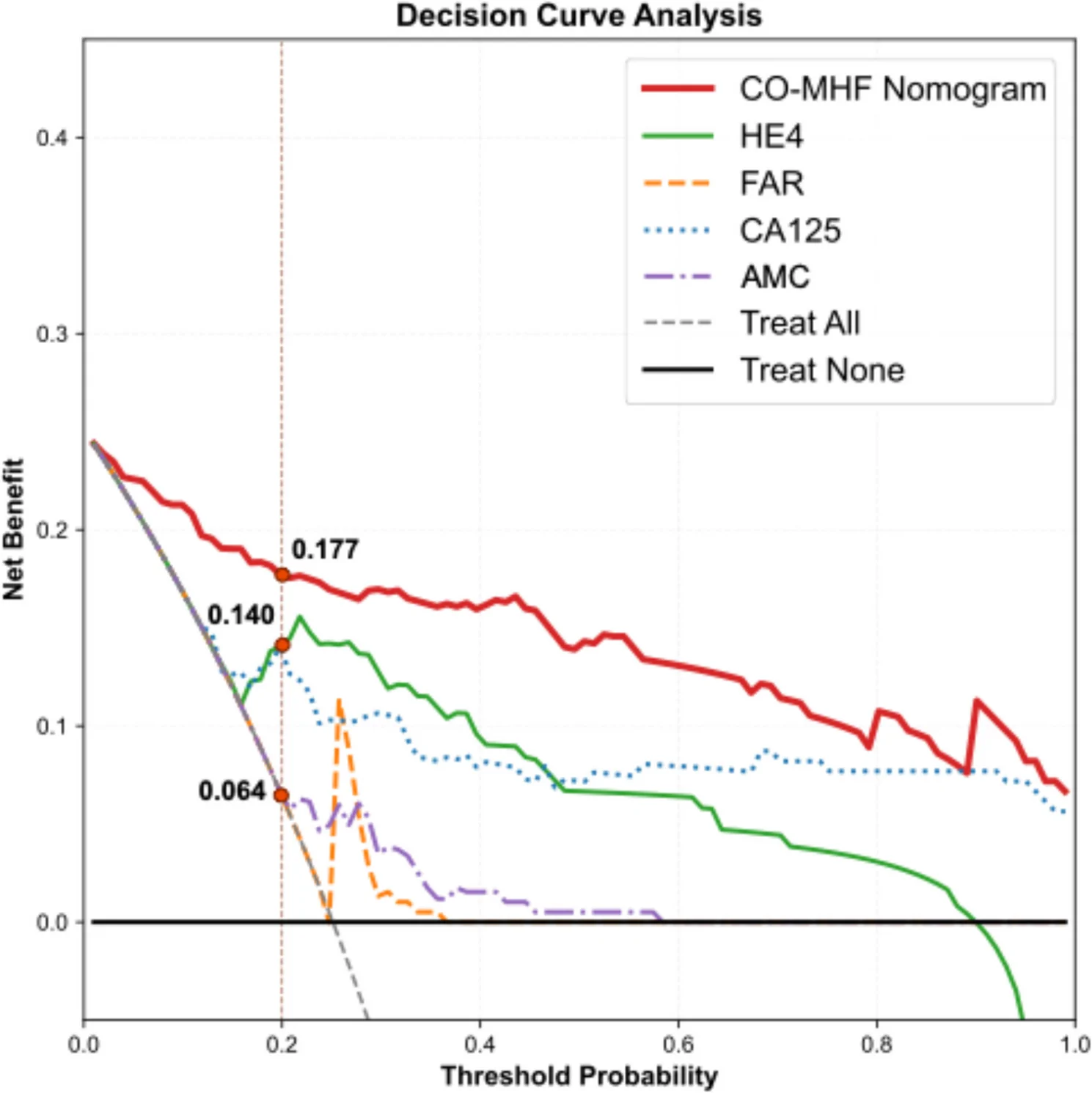
Decision curve analysis evaluating the clinical net benefit of the predictive model. The composite nomogram showed favorable net benefit across a broad continuum of threshold probabilities compared with individual diagnostic indicators and treat-all or treat-none baseline strategies

DCA was performed to evaluate the clinical utility of the CO-MHF nomogram (Fig. 6). The composite model showed greater net benefit than the treat-all or treat-none strategies across clinically relevant threshold probabilities. At a 20% threshold probability, the CO-MHF nomogram provided a net benefit of 0.177, exceeding the net benefit of 0.140 observed for both HE4 and CA125, as well as the lower net benefit values produced by FAR and AMC.

## Discussion

Preoperative differentiation between benign OE and EAOC remains clinically important because misclassification may lead either to overtreatment of benign disease or to inadequate staging of occult malignancy [22, 23]. Given the clinical burden and biological heterogeneity of ovarian cancer, risk stratification based only on single tumor-antigen markers is unlikely to fully capture malignant transformation [24, 25]. In this study, we developed and internally evaluated the CO-MHF model as a multidimensional preoperative risk tool integrating systemic immune-coagulation indicators with conventional tumor markers and clinical variables. These findings suggest that combining tumor-antigen markers with host-response markers may improve preoperative risk assessment in a setting where benign inflammation and malignant transformation frequently overlap.

The present findings should be interpreted within the evolving framework of EAOC heterogeneity. Recent studies have distinguished endometriosis-correlated ovarian carcinoma, characterized by direct histopathological transition from endometriosis to carcinoma, from endometriosis-incidental ovarian carcinoma, in which endometriosis is present without identifiable transitional lesions [7, 8]. This distinction supports the concept that EAOC is not a single uniform disease entity. Although our cohort was designed to distinguish EAOC from benign OE rather than to subclassify EAOC into endometriosis-correlated and endometriosis-incidental entities, the observed benefit of integrating systemic host-response markers with tumor markers is consistent with the need for multidimensional risk assessment in a heterogeneous disease spectrum. Future studies should evaluate whether CO-MHF performs differently across these EAOC subtypes.

The biological rationale of the CO-MHF model is supported by the role of FAR and AMC as accessible indicators of systemic host response. Malignant progression may promote inflammatory and coagulation remodeling, including increased fibrinogen synthesis, reduced albumin levels, and monocyte recruitment into the tumor microenvironment [26, 27]. FAR integrates hypercoagulability, inflammation, and nutritional depletion, whereas AMC may partly reflect monocyte-macrophage activation. These features may complement CA125 and HE4, which mainly reflect tumor-associated antigen release. In the present cohort, CA125 alone had a PPV of 53.0%, indicating a substantial false-positive burden in benign inflammatory disease. By contrast, the CO-MHF model achieved a PPV of 86.0% and a specificity of 95.9%, suggesting that systemic host-response markers may help reduce benign inflammatory noise in the preoperative assessment of EAOC-related ovarian masses [28, 29].

The supplementary data-driven analyses provided empirical support for the prespecified CO-MHF predictor set. AIC-based stepwise logistic regression selected the same variables as the CO-MHF model, and LASSO regression retained all CO-MHF predictors. Although NLR was additionally selected by LASSO, its incremental contribution was limited and it was not retained by AIC-based selection. Age was retained as an empirically supported clinical adjustment variable because removing age reduced discrimination and worsened model fit; however, its coefficient should be interpreted as adjustment within an age-overlapping and menopause-adjusted diagnostic cohort rather than as evidence of an independent biological protective effect. AMC showed a smaller marginal contribution than FAR, HE4, CA125, and menopausal status, but was retained as a biologically coherent immune-response component reflecting monocyte-macrophage activation. These findings suggest that the CO-MHF predictor set was supported by both biological rationale and supplementary data-driven assessment.

The Youden-derived threshold of 0.468 should be interpreted as an accuracy-optimized reference point rather than a mandatory clinical decision boundary. At this threshold, the model achieved high specificity but a sensitivity of 75.5%, indicating that some EAOC cases could still be missed. We therefore evaluated additional sensitivity-prioritized thresholds. Lowering the cut-off to 0.167 increased sensitivity to 85.7% and NPV to 94.5%, while lowering it further to 0.099 increased sensitivity to 93.9% and NPV to 97.3%, with corresponding reductions in specificity. These findings indicate that CO-MHF should be used as a continuous risk assessment tool, with threshold selection adapted to the clinical objective. A higher-specificity threshold may be useful when avoiding overtreatment is prioritized, whereas a lower threshold may be preferable when minimizing missed malignancy and ensuring appropriate surgical staging are the primary concerns. This threshold-dependent interpretation is also supported by DCA, which showed favorable net benefit across a broad range of threshold probabilities.

Several limitations should be acknowledged. First, the number of EAOC cases was relatively small (*n* = 49), reflecting the rarity of EAOC and the strict eligibility criteria. The final cohort also showed an approximate 1:3 imbalance between EAOC and OE cases. We did not apply artificial over-sampling or under-sampling in the primary model because the goal was to preserve probability calibration for an interpretable clinical nomogram rather than to optimize a purely classification-oriented algorithm. To limit data-driven overfitting, we retained the primary CO-MHF model as a clinically interpretable, mechanism-informed logistic-regression nomogram. Supplementary LASSO regression, AIC-based stepwise selection, and marginal contribution analyses were added to empirically evaluate the selected predictor set, but these analyses were interpreted as supportive rather than definitive because the number of EAOC events was limited. In addition, class-weighted logistic regression using the same predictors yielded an AUC comparable to that of the primary model, supporting the robustness of the main findings under the observed class distribution. Nevertheless, multivariable modeling in a limited retrospective cohort remains vulnerable to coefficient instability and overfitting.

Second, the study had a substantial attrition rate, with 650 of 845 screened patients excluded before construction of the final analytical cohort. This selection strategy was intended to reduce confounding from non-tumor-related inflammatory, immune, and coagulation disturbances, because FAR and AMC may be affected by infection, autoimmune disease, benign inflammatory gynecological conditions, organ dysfunction, and medications affecting coagulation or immune profiles. However, this approach reduced sample size and may have limited generalizability. In particular, the exclusion of 225 patients because of incomplete preoperative laboratory data may have introduced attrition bias. Because the study covered a 10-year period, HE4 testing for apparently benign ovarian masses was gradually adopted in clinical practice. Patients from earlier years or those with lower suspicion of malignancy may therefore have been less likely to undergo HE4 testing and consequently less likely to meet the complete-predictor requirement for the CO-MHF model. Accordingly, the performance of CO-MHF should be interpreted as applicable mainly to patients with complete preoperative coagulation, inflammatory, nutritional, and tumor-marker profiles.

Third, the final analytical cohort should not be interpreted as reflecting the institutional prevalence or proportional distribution of EAOC among all ovarian cancers. During the same study period, 546 patients underwent surgery for primary ovarian malignancies at our institution, including 482 patients with epithelial ovarian cancer and 76 patients with histopathologically confirmed EAOC. The final EAOC-versus-OE cohort was deliberately constructed for the diagnostic task of distinguishing EAOC from benign OE under complete preoperative laboratory availability. Thus, the enriched proportion of EAOC cases reflects diagnostic model construction rather than the background frequency of EAOC in the general ovarian cancer population.

Fourth, the benign OE controls in the final analytical cohort were older than the general population of patients with OE. This reflects the diagnostic objective of distinguishing EAOC from benign OE in a clinically ambiguous, age-overlapping surgical population, rather than describing the full epidemiological age distribution of benign OE. No strict individual age matching was performed; therefore, age was retained as a prespecified clinical covariate in the CO-MHF model. We acknowledge that a separate analysis of younger OE patients would further contextualize the broader benign OE population. However, reliable subgroup analysis of younger OE patients was not feasible in this retrospective dataset because many younger OE candidates did not have uniformly complete preoperative tumor-marker, coagulation, inflammatory, and nutritional profiles required for the CO-MHF framework. Therefore, the model should not be interpreted as a broad screening tool for all reproductive-age patients with typical OE, and prospective validation in younger OE populations is warranted.

Finally, the absence of independent external validation remains an important limitation. Prediction models developed in retrospective single-center cohorts may be affected by selection bias, spectrum effects, and local testing practices [30]. External validation across diverse populations is required to confirm that the model does not overfit the specific characteristics of the derivation cohort [31, 32]. Therefore, although the biological rationale, calibration assessment, DCA, threshold exploration, and sensitivity analyses support CO-MHF as a candidate tool for preoperative risk stratification, its clinical role should be confirmed through prospective multicenter validation, particularly in broader OE populations, younger reproductive-age patients, and external cohorts with standardized laboratory testing. Together with prior EAOC modeling studies [33], these findings should be viewed as hypothesis-generating until externally validated.

## Conclusion

In this cohort, systemic immune and coagulation markers, represented by the FAR and AMC, were associated with improved discrimination between EAOC and benign OE when combined with HE4, CA125, and key clinical factors. The resulting CO-MHF model showed favorable internal diagnostic performance and net clinical benefit across clinically relevant decision thresholds. Supplementary data-driven analyses, including LASSO regression, AIC-based stepwise selection, and marginal contribution assessment, also supported the selected predictor set and suggested limited additional diagnostic value from excluded candidate variables. These findings support a practical, mechanism-informed approach to preoperative risk stratification for EAOC-associated ovarian masses, although prospective multicenter external validation and clinical threshold optimization remain necessary before clinical implementation.

## Supplementary Information

Supplementary Material 1: Supplementary Figure 1. Relative contributions of predictors in the final CO-MHF logistic-regression model based on standardized regression coefficients. The visualization elucidates the mathematical weighting of demographic factors and systemic indices alongside tumor load markers.

Supplementary Material 2: Supplementary Table S1. Source population, diagnostic cohort construction, and exclusion reasons. Supplementary Table S2. Data-driven predictor selection and marginal contribution analysis for the CO-MHF model. Supplementary Table S3. Threshold-dependent diagnostic performance of the CO-MHF model. Supplementary Table S4. Sensitivity analysis addressing the moderate EAOC-to-OE class imbalance.

## Data Availability

The datasets used and/or analyzed during the current study are available from the corresponding authors on reasonable request.

## Abbreviations

AIC: Akaike information criterion
AMC: Absolute monocyte count
AUC: Area under the receiver operating characteristic curve
CA125: Carbohydrate antigen 125
CI: Confidence interval
CO-MHF: Composite model incorporating CA125, clinical factors, monocytes, HE4, and FAR
DCA: Decision curve analysis
EAOC: Endometriosis-associated ovarian cancer
FAR: Fibrinogen-to-albumin ratio
HE4: Human epididymis protein 4
IQR: Interquartile range
LASSO: Least absolute shrinkage and selection operator
NLR: Neutrophil-to-lymphocyte ratio
NPV: Negative predictive value
OE: Ovarian endometrioma
PPV: Positive predictive value
ROC: Receiver operating characteristic
