# Supplementary material for: Systemic immune and coagulation amplifiers resolve diagnostic ambiguity in endometriosis-associated ovarian cancer
Source: J Ovarian Res. 2026 Jul 23;19:319. doi: 10.1186/s13048-026-02205-8 (PMC13681925; doi:10.1186/s13048-026-02205-8)
Supplement: Supplementary file 1 [file 13048_2026_2205_MOESM1_ESM.docx]

**Supplementary Figure 1.** **Relative contributions of predictors in the final CO-MHF logistic-regression model based on standardized regression coefficients.** The visualization elucidates the mathematical weighting of demographic factors and systemic indices alongside tumor load markers.
