# Supplementary material for: Systemic immune and coagulation amplifiers resolve diagnostic ambiguity in endometriosis-associated ovarian cancer
Source: J Ovarian Res. 2026 Jul 23;19:319. doi: 10.1186/s13048-026-02205-8 (PMC13681925; doi:10.1186/s13048-026-02205-8)
Supplement: Supplementary file 2 [file 13048_2026_2205_MOESM2_ESM.docx]

**Supplementary Table S1.** Source population, diagnostic cohort construction, and exclusion reasons.

| **Category** | **No. of patients** | **Percentage** |
| --- | --- | --- |
| ***Institutional ovarian malignancy denominator*** | | |
| Primary ovarian malignancies during the study | 546 | 100.0% |
| Epithelial ovarian cancer | 482 | 88.3% of ovarian malignancies |
| Histopathologically confirmed EAOC | 76 | 15.8% of epithelial ovarian cancer |
| ***EAOC-versus-OE diagnostic source cohort*** | | |
| Patients screened for the EAOC-versus-OE diagnostic model | 845 | 100.0% |
| EAOC candidates | 76 | 9.0% of screened cohort |
| Benign OE candidates | 769 | 91.0% of screened cohort |
| ***Exclusion and final analytical cohort*** | | |
| Excluded patients | 650 | 76.9% of screened cohort |
| Incomplete preoperative laboratory data | 225 | 26.6% of screened cohort |
| Concomitant malignancies or metastatic tumors | 18 | 2.1% of screened cohort |
| Concomitant benign gynecological conditions affecting CA125 | 142 | 16.8% of screened cohort |
| Acute inflammatory conditions or autoimmune diseases | 74 | 8.8% of screened cohort |
| Recent medications affecting coagulation or immune profiles | 191 | 22.6% of screened cohort |
| Final analytical cohort | 195 | 23.1% of screened cohort |
| EAOC included in final cohort | 49 | 25.1% of final cohort |
| Benign OE included in final cohort | 146 | 74.9% of final cohort |
| Note: Percentages were calculated using the denominator specified in each section. The institutional ovarian malignancy denominator was used to clarify the background source population. The EAOC-versus-OE diagnostic source cohort was constructed for model development and should not be interpreted as estimating the prevalence of EAOC among all ovarian cancers. Each excluded patient was assigned to the primary exclusion category used in the screening flowchart. | | |

**Supplementary Table S2.** Data-driven predictor selection and marginal contribution analysis for the CO-MHF model.

| **Candidate predictor** | **LASSO selected** | **AIC stepwise selected** | **Marginal contribution analysis** | **Final decision and interpretation** |
| --- | --- | --- | --- | --- |
| Age | ✓ | ✓ | Removing age reduced AUC from 0.932 to 0.918 and worsened AIC from 118.44 to 130.07. | Retained as an empirically supported clinical adjustment variable rather than interpreted as an independent biological driver. |
| Menopausal status | ✓ | ✓ | Selected by both data-driven procedures and incorporated in the final CO-MHF model. | Retained as a major clinical predictor reflecting menopause-related clinical risk context. |
| AMC | ✓ | ✓ | Removing AMC reduced AUC from 0.932 to 0.927. | Retained as a biologically coherent immune-response component with modest incremental contribution. |
| CA125 | ✓ | ✓ | Selected by both data-driven procedures and incorporated in the final CO-MHF model. | Retained as a conventional tumor-antigen marker with empirical support in the data-driven assessment. |
| HE4 | ✓ | ✓ | Removing HE4 reduced AUC from 0.932 to 0.923. | Retained as a clinically established ovarian malignancy marker with empirical support in the data-driven assessment. |
| FAR | ✓ | ✓ | Removing FAR reduced AUC from 0.932 to 0.916. | Retained as a host-response marker integrating coagulation activation and albumin-related inflammatory or nutritional status. |
| NLR | ✓ | — | Adding NLR to the CO-MHF model increased AUC only slightly from 0.932 to 0.936 without improving model parsimony. | Not retained because its incremental diagnostic gain beyond the final CO-MHF predictor set was limited. |
| Pregnancy history | — | — | No meaningful incremental contribution was observed in the data-driven candidate predictor assessment. | Not retained because its additional diagnostic value was limited in the present complete-case cohort. |
| Fibrinogen | Not entered | Not entered | Represented by FAR to avoid mathematical redundancy and multicollinearity. | Not separately retained; its information was incorporated through FAR. |
| Albumin | Not entered | Not entered | Represented by FAR to avoid mathematical redundancy and multicollinearity. | Not separately retained; its information was incorporated through FAR. |
| Note: The primary CO-MHF model included age, menopausal status, AMC, CA125, HE4, and FAR. LASSO logistic regression and AIC-based stepwise logistic regression were performed as supplementary data-driven predictor-selection analyses using a non-redundant candidate predictor set. “✓” indicates that the candidate predictor was selected by the corresponding method, whereas “—” indicates that the candidate predictor was not selected. Because FAR is mathematically derived from fibrinogen and albumin, fibrinogen and albumin were not entered simultaneously with FAR in the primary candidate set to reduce redundancy and multicollinearity. Add-one and drop-one marginal contribution analyses were performed to evaluate the incremental value of retained and excluded candidate variables based on changes in AUC and AIC. LASSO additionally selected NLR; however, NLR was not selected by AIC-based stepwise regression and provided only a minimal AUC increase when added to the CO-MHF model. Therefore, NLR was not incorporated into the final nomogram. | | | | |

**Supplementary Table S3.** Threshold-dependent diagnostic performance of the CO-MHF model.

| **Threshold strategy** | **Cut-off** | **Sensitivity** | **Specificity** | **Accuracy** | **PPV** | **NPV** | **Clinical interpretation** |
| --- | --- | --- | --- | --- | --- | --- | --- |
| Youden-derived threshold | 0.468 | 75.5% | 95.9% | 90.8% | 86.0% | 92.1% | Accuracy-optimized threshold |
| Sensitivity-prioritized threshold | 0.167 | 85.7% | 82.2% | 83.1% | 61.8% | 94.5% | Reduced false-negative risk |
| Rule-out-oriented threshold | 0.099 | 93.9% | 74.7% | 79.5% | 55.4% | 97.3% | Higher sensitivity for oncologic preparedness |
| Note: The Youden-derived threshold was used as the primary accuracy-optimized cut-off. Lower thresholds were explored to illustrate the trade-off between improved sensitivity and reduced specificity when minimizing false-negative classification is clinically prioritized. | | | | | | | |

**Supplementary Table S4.** Sensitivity analysis addressing the moderate EAOC-to-OE class imbalance.

| **Model** | **Strategy** | **AUC** | **Sensitivity** | **Specificity** | **Accuracy** | **PPV** | **NPV** |
| --- | --- | --- | --- | --- | --- | --- | --- |
| CO-MHF model | Unweighted logistic regression | 0.932 | 75.5% | 95.9% | 90.8% | 86.0% | 92.1% |
| Sensitivity model | Class-weighted logistic regression | 0.936 | 77.6% | 95.9% | 91.3% | 86.4% | 92.7% |
| Note: The class-weighted model was used only as a sensitivity analysis to evaluate the robustness of model discrimination under the observed class imbalance. It was not used to replace the primary calibrated CO-MHF nomogram. | | | | | | | |
